# 4065 Preferences, Expectancies, and Stigma among Treatment Seeking Combat PTSD Patients

**DOI:** 10.1017/cts.2020.140

**Published:** 2020-07-29

**Authors:** John Moring, Alan Peterson, Casey Straud, Jim Mintz, Paul Nabity, Lindsay Bira, Stacey Young-McCaughan, Willie Hale, Donald McGeary, Patricia Resick

**Affiliations:** 1 University of Texas Health Science Center San Antonio; 2 UT Health San Antonio; 3 University of Texas at San Antonio; 4 Duke University Medical Center

## Abstract

OBJECTIVES/GOALS: Cognitive Processing Therapy (CPT) is a cognitive behavioral treatment for posttraumatic stress disorder (PTSD). CPT is effective in treating combat-related PTSD among Veterans and active duty service members. It is unknown whether improvement in PTSD is related to accommodation of patient preference of the modality of therapy, such as in-office, telehealth, and in-home settings. An equipoise-stratified randomization design allows for complete randomization of participants who are interested and eligible for all three treatment arms. It also allows participants to reject one treatment arm if they are not interested or eligible. Participants who elect to opt out of one arm are randomized to one of the two remaining treatment arms. The primary aim of this study was to evaluate differences in patient satisfaction, treatment stigma beliefs, and credibility beliefs based on patient treatment modality preference. The second aim of this study was to examine if baseline satisfaction, stigma beliefs, and credibility beliefs predicted PTSD treatment outcomes. METHODS/STUDY POPULATION: Active duty service members and veterans with PTSD (N = 123) were randomized to one of three arms using an equipoise stratified randomization. Participants underwent diagnostic interviews for PTSD at baseline and post-treatment and completed self-report measures of satisfaction, stigma, credibility and expectancies of therapy. RESULTS/ANTICIPATED RESULTS: A series of ANOVAs indicated that there were group differences on patient stigma beliefs regarding mental health, F = 5.61, p = .001, and therapist credibility, F = 5.11, p = .002. Post hoc analyses revealed that participants who did not opt of any treatment arm demonstrated lower levels of stigma beliefs compared to participants who opted-out of in-office, p = .001. Participants who opted out of in-home viewed the therapist as less credible compared to participants who did not opt of any arm, p = .004. Multiple regression analysis found that baseline patient satisfaction, stigma beliefs, and credibility beliefs were not predictive of PTSD treatment outcomes, p > .05. DISCUSSION/SIGNIFICANCE OF IMPACT: Combat PTSD patients may opt out of in-office therapy due to mental health stigma beliefs, and visibility in mental health clinics may be a concern. For patients who opted out of in-home therapy, lack of credibility may have decreased participants’ desire for therapists to enter their home. Despite concerns of mental health stigma and the credibility of the therapy in certain treatment arms, patients in each treatment arm significantly improved in PTSD symptomotology. Moreover, patient characteristics, including satisfaction, stigma, and credibility of the therapy, did not significantly predict treatment outcomes, which demonstrates the robustness of Cognitive Processing Therapy.

